# Highly Integrated Cladding Mode Stripper Array for Compact High-Power Industrial Fiber Laser

**DOI:** 10.3390/mi13122226

**Published:** 2022-12-15

**Authors:** Yu Liu, Wenjie Wu, Pengfei Zhao, Shan Huang, Yuwei Li, Yue Li, Min Li, Rumao Tao, Honghuan Lin, Jianjun Wang

**Affiliations:** Laser Fusion Research Center, China Academy of Engineering Physics, Chengdu 610200, China

**Keywords:** cladding mode stripper, fiber laser, integrated device, optical fiber device, compact laser

## Abstract

A design integrating multiple cladding mode strippers used in fiber laser architectures into a single device is proposed. This approach can increase the compactness of fiber lasers, thus contributing to industrial laser processing applications. By offset-placing the most intense light-stripping parts, for instance, by inversing the laser injection directions or by displacing the beginning of etched sections, multiple cladding mode strippers bundled together into a single housing can have the hottest regions separated and can operate at full power simultaneously, with no evident cross-influence on each other. Two and three cladding-mode-stripper arrays have been implemented, and validation tests have been conducted with ~500-W cladding power being injected into each input port. For both arrayed devices, compared to the scenario in which only a single cladding mode stripper is working, no greater than a 2.1 °C temperature increment is generated when all components are operating concurrently, which demonstrates the effectiveness of the integration method. In this way, one half and two thirds of space/weight reduction can be realized, respectively, for the two and three cladding-mode-stripper arrays, which is meaningful, since cladding mode strippers are among the most bulky and hottest components in fiber lasers. Moreover, this integration provides a valuable reference for the miniaturization of other components, and thus, could contribute to the development fiber lasers with higher power-to-volume ratios, which would be more economical for industrial applications.

## 1. Introduction

Fiber lasers have been developed and utilized in the material processing industry in recent years because of their high wall-plug efficiency, easy maintenance, small footprint and the merits of fiber beam delivery [1,2,3,4,5]. For commercial industrial fiber lasers, compacter size and lighter weight are important development directions, as they can save valuable space and reduce operational costs [6,7,8,9]. Therefore, it is important for laser architectures, including the constituent fiber material and fiber components, to be small and tightly arranged. In fact, optical fiber is quite flexible; as such, it can be coiled into a compact spool, while on the other hand, fiber components usually occupy a considerable amount of space for mechanical protection and thermal dissipation purposes, especially in high power applications. Therefore, the integration and miniaturization of these devices play an important role in the development of new fiber lasers which better suit the demands of industrial applications.

The cladding mode stripper (CMS) [10,11,12,13,14,15,16,17,18,19,20] is an indispensable component in fiber laser systems, as it removes the cladding light in the fiber, i.e., mainly pump light residue and signal light leakage. In order to clean the cladding power as much as possible and to avoid severe thermal issues caused by abrupt light leakage, high power CMSs are at least many centimeters [18,19,20] or even several meters in length [16]. Additionally, due to the great amount of optical radiation, high power CMSs generally require water-cooled housing to block the light and to dissipate the optical-induced heat load [13,14,15,16,17,19]. Therefore, the CMS device including its housing is indeed one of the bulkiest components in fiber laser systems; this puts a limitation on laser footprint reduction. The typical architecture of master oscillator power amplifiers (MOPAs) contains several CMSs, e.g., one between the oscillator/amplifier stages and one before the final output. The total volume of these CMS devices is the volume of a single CMS multiplied by the number of CMSs used; the area required becomes even larger when the cold plate and engine cabinet are included, and the power-to-volume ratio of such lasers can be unsatisfactory.

Research efforts on CMSs have been mainly focused on developing innovative fabrication techniques [10,11,12,13,14,17] or improving the power handling [15,16,18,19,20] and power attenuation [18,20] abilities. Only a few papers [19] have mentioned the miniaturization of CMS devices, which is a key aspect from a laser engineering viewpoint.

In this work, a design to integrate multiple CMSs into a single device is proposed. The integrated CMS has N input ports and N output ports; thus, it is designated as an N-CMS array. Using the housing of a home-made 500-W CMS, of which the length, width and height are 20 cm, 2 cm and 2 cm, respectively, two-CMS and three-CMS integrations have been implemented and up to 1.5-kW full-power functionalization has been demonstrated, saving one half and two thirds, respectively, in both space and weight. With this design, the total volume of the CMSs in fiber lasers could be reduced to that of a single CMS.

## 2. Design and Development

**Figure 1:**
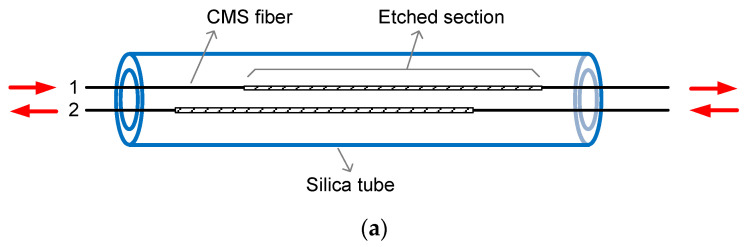

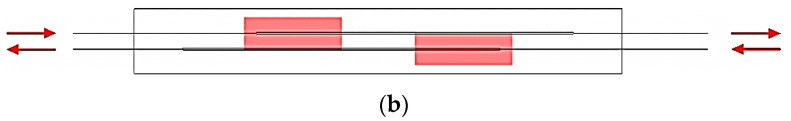
(**a**) Schematic diagram of the two-CMS array and (**b**) the distribution of the hot regions.

Individual CMSs have been fabricated by applying chemical etchant on the cladding surface of double-cladding fibers (DCF). According to previous investigations [19], in spite of the rather long length, i.e., 20 cm, applied to thoroughly deplete the cladding light, light leakage is actually not uniformly distributed along the whole etched length, but rather, is concentrated in the first 2 cm, as is the heat load. Based on these findings, it is reasonable to think that, with the hottest regions being staggered, multiple CMSs could be integrated into a single device without causing significant heat accumulation. To prove this idea, a two-CMS integrated device—two-CMS array—has been made, for which schematic drawings are shown in Figure 1a, with the red arrows indicating the propagation directions of the cladding laser within each fiber. The fabrication details are as follows. The CMS fibers were 25/400 μm DCF and lengths of 18 cm were coating-stripped. Regarding the coating edge closer to the laser injection end to be the origin position, both fibers were etched from the 4th cm to the 17th cm on the coating-free part. After that, the fibers were bundled together and inserted through a 20-cm long, 1.2/3.0-mm inner/outer-diameter fused-silica tube, with the respective input and output fiber ends being placed in opposite configurations. In this way, the hottest regions of the two CMSs should be around 8 cm from each other in principle, as Figure 1b indicates.

**Figure 2:**
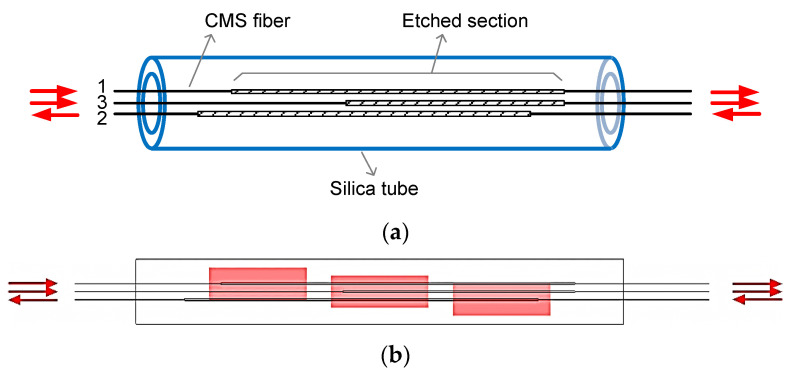
(**a**) Schematic diagram of the three-CMS array and (**b**) the distribution of the hot regions.

The two-CMS array can be applied to a MOPA system consisting of one amplifier stage. For more general MOPA laser types, and narrow linewidth fiber lasers in particular, multiple amplifier stages are employed; therefore, more than two CMSs have to be used. In order to increase the number of CMSs integrated in a single device, apart from inversing the etched sections, it is also proposed to offset the beginning of the etched sections, as shown in the sketch of a three-CMS array in Figure 2a. To make the three-CMS integrated device, 25/250 μm DCF were used and the lengths of the stripped windows were 18 cm. Two fibers were etched from 2.5 to 17 cm, the third one was etched from 7.5 to 17 cm, and the three CMS fibers were bundled with the bare parts sealed inside a 20-cm long fused-silica tube. It is noteworthy that the first and the third CMS fibers were placed so as to have the same laser direction, while the second CMS fiber was positioned in the opposite way. Therefore, the hottest regions of the three CMSs should be separated from each other by a distance of 5.5 cm, as Figure 2b shows.

## 3. Experiment Results and Discussion

**Figure 3:**
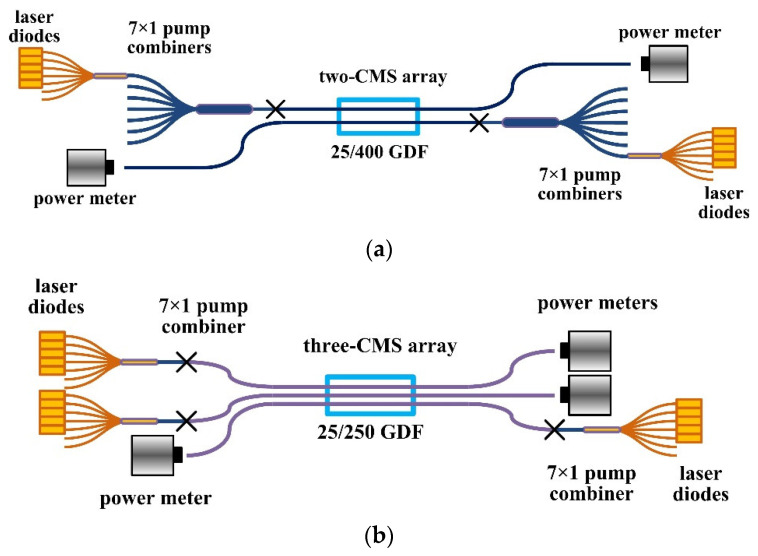
Experimental setup for measuring (**a**) the two-CMS array and (**b**) the three-CMS array.

In order to work safely at high power, CMS arrays were further packaged in water-cooled aluminum housings, of which the length × width × height were 20 cm × 2 cm × 2 cm. Silica tubes were fully enclosed in the housings so that the stripped light would be completely blocked and absorbed. Then, the performance of the CMS arrayed devices was investigated by building a measurement system, as shown in Figure 3. The input port of each CMS was injected with about 500 W of cladding power, provided by one set—four pieces—of 915-nm laser diodes (LDs). For the two-CMS array, two cascaded 7 × 1 fiber combiners were used to connect between the LDs and the CMS, while for the three-CMS array, only the first 7 × 1 fiber combiner was employed. The terminating fibers of the two combiners were 220/242 μm multimode fiber and 25/400 μm GDF, with the numerical aperture (NA) being 0.22 and 0.46, respectively. In this way, the fiber splices between the combined LDs and the CMS devices were symmetric and easy to implement. It should be pointed out that three sets of LDs were adopted in the experiments: one was with 95% of the power confined in 0.16 of NA—type I, while the other two sets were with 95% of the power confined in 0.14 of NA—type II. The two-CMS array was tested using one set of type I LDs and one set of type II LDs, while the three-CMS array was tested using one set of type I LDs and two sets of type II LDs. It is also worth noting that during the experiments, the flux and temperature of the water coolant provided to the CMS housing were about 9.4 L/min and 22.5 °C, respectively.

**Figure 4:**
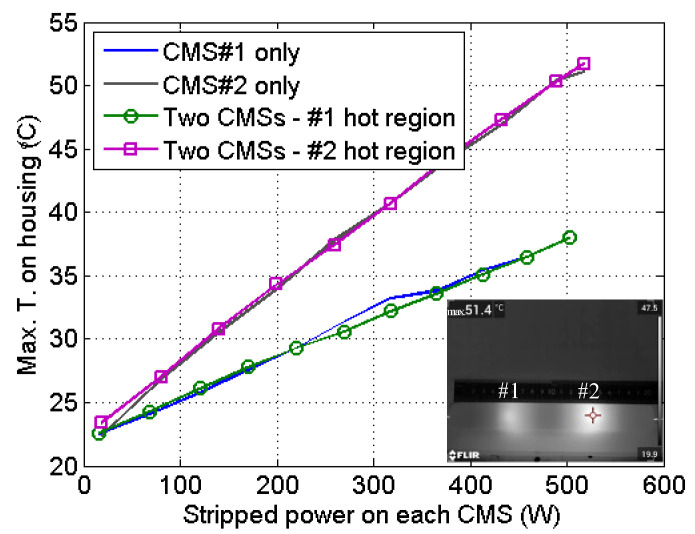
Thermal image and temperature behavior of the two-CMS array.

The experimental results of the thermal image and temperature behavior of the two-CMS array are summarized in Figure 4. As shown, the hottest regions of the integrated CMSs were located at different positions, about 8 cm from one another, which is consistent with the intended values. As such, barely any cross thermal influence was observed. This is further evidenced in the temperature–power curves. The solid lines correspond to the case when only one CMS was operating, while the marked lines are the maximum temperatures at the respective hottest regions when both CMSs were working. It is clear that the solid lines and the marked lines coincide well. For instance, the maximum temperature on the housing was 51.1 °C when CMS#1 was receiving ~500 W of power and 38.0 °C when CMS#2 was receiving ~500 W of power. When the two-CMS array was fully operational (CMS#1 and CMS#2 simultaneously receiving ~500 W of power and the housing dissipating ~1000 W of power), the maximum temperatures at the hottest regions were 51.7 °C and 38.0 °C, showing little variation compared to when a single CMS was active. We may therefore conclude that integrating two CMSs using the proposed method does not significantly increase the heat density, allowing both CMSs operate safely but providing the advantage of a 50% reduction in both volume and weight. Here, it is worth noting that the temperature difference between CMS#1 and CMS#2 was due to the difference in LD brightness; the brighter the LD, the cooler the CMS. Apart from the thermal behavior, the power attenuation was also measured; the values were 99.79% and 99.60% for CMS#1 and CMS#2, respectively.

**Figure 5:**
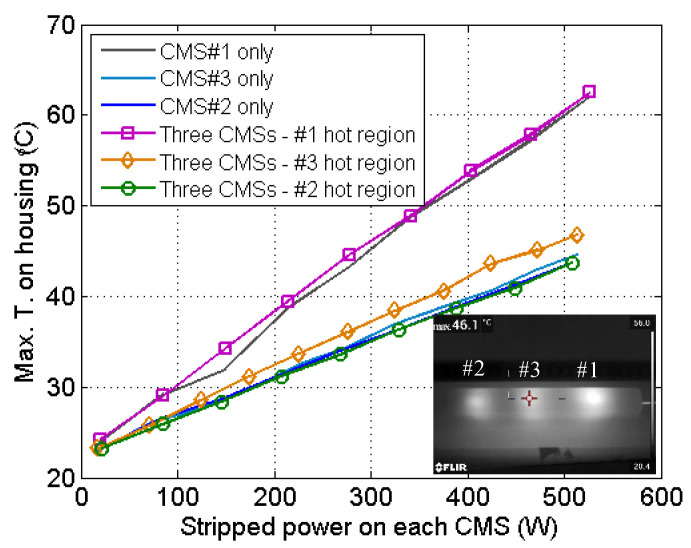
Thermal image and temperature behavior of the three-CMS array.

As shown in Figure 5, the three-CMS array behaved similarly. The hottest regions of the three CMSs were separated by ~6 cm, with a low degree of influence on each other. In the case the three CMSs working separately, the maximum temperatures on the housing were 62.0 °C, 43.7 °C, and 44.7 °C, respectively, at ~500 W of stripped power. Meanwhile, with the three CMSs operating together (i.e., with ~1500 W of total power being dissipated in the housing), the maximum temperatures at the hottest regions were 62.5 °C, 43.8 °C, and 46.8 °C. On this basis, it was observed that CMS#1 and CMS#2 behaved in almost the same was, while CMS#3—the one located in the center—exhibited a 2.1-°C temperature increment due to double heat accumulation from the two adjacent CMSs. Again, this experiment validated the effectiveness of integrating three CMSs using the proposed method. The heat density barely increased, so that all three CMSs could safely operate with the volume being 1/3 of the original value. The temperature of CMS#1 was evidently higher than those of the other two because LDs of inferior NA were used. The power attenuations of CMS#1, CMS#2, and CMS#3 were 99.18%, 99.23%, and 99.07%, respectively. CMS#3 exhibited lower stripping efficiency because the etched section was 5 cm shorter.

By comparing Figure 4 and Figure 5, one can also find that the three-CMS array exhibited higher temperatures than the two-CMS array; this was due to the smaller diameter of the fiber than that used in the three-CMS array. With the same amount of cladding power, fiber with a smaller diameter will induce higher power density on the cladding surface, which, in turn, will generate a greater stripping rate and thermal density at the beginning of the etched section. Additionally, it was clear that the attenuations of the three-CMS array were lower than those of the two-CMS array; the main reason for this lies in the difference of NA of the combiner delivery fiber. It is known that lower-NA light is less sensitive to the CMS stripping structure [10]; therefore, the three-CMS array performed less well. Furthermore, it can be observed from the thermal image in Figure 5 that a section of the housing did not become bright, which means that more CMSs could be added. For instance, two more CMSs could be integrated, with one radiating the interval between hot regions #1 and #3 and the other heating the interval between regions #2 and #3. This implementation would be very useful for narrow-linewidth fiber lasers, since the architecture typically contains three or more amplifier stages, so that at least five CMSs are needed.

## 4. Conclusions

A method to integrate multiple CMSs in a single device is proposed. By inversing and/or displacing the etched sections of the CMSs, the hottest regions can be staggered, and the heat density is almost the same as when only one CMS is active. Using this design, two- and three-CMS arrays have been fabricated, respectively demonstrating full-power stripping abilities of ~1000 W and ~1500 W. Both the size and weight have been effectively reduced compared to traditional designs, and no evident heat accumulation or heat cross-influence were observed. With such a design, the MOPA structure of fiber lasers can be realized in a more compact way, since it has been shown that the bulkiest components can be organized to form a single unit. Further, by offsetting more CMSs, N-CMS arrays with increased N can also be implemented. This report provides a valuable reference for fiber laser engineering and industrial laser upgrades.

## Data Availability

Not applicable.
